# Autism Spectrum Disorder: The Cerebellum, Genes, and Pathways

**DOI:** 10.3390/neurolint17100173

**Published:** 2025-10-14

**Authors:** Santosh R. D’Mello

**Affiliations:** 1 College of Arts and Sciences, Louisiana State University, Shreveport, LA 71103, USA; dmello@neugeneron.com; Tel.: +1-214-909-3443; 2 Center for Molecular Neuroscience, Kasturba Medical College, Manipal Academy of Higher Education, Manipal 576104, Karnataka, India; 3 Neugeneron LLC., Dallas, TX 75243, USA

**Keywords:** autism, cerebellum, Purkinje neurons, genes, signaling pathways, synaptic dysfunction

## Abstract

Autism spectrum disorder (ASD) is a complex, heterogenous, and prevalent neurodevelopmental disorder characterized by core symptoms, including social communication deficits, restrictive interests, and repetitive behaviors. Although environmental factors contribute to the etiology of ASD, the disorder has a strong genetic basis, although the specific genes involved in causing or contributing to the disorder remain to be conclusively identified. Whereas previous studies have focused on the cerebral cortex, hippocampus, and associated brain regions to uncover the underpinnings of ASD, emerging evidence indicates that dysfunction of the cerebellum is one of the most consistent associates of ASD. Traditionally thought to function solely in motor control, more recent studies have established that projections from the cerebellum make mono- and polysynaptic connections to a variety of non-motor areas including the cerebral cortex, hypothalamus, and hippocampus, and is involved in a range of cognitive, sensory, and behavioral functions. While several reviews of the molecular underpinnings of ASD have focused on the other brain regions, primarily the cortex, in this review we describe the key role that the cerebellum plays in the development of ASD and then focus on genetic variations that cause ASD, focusing on genes expressed and studied in the cerebellum. We have divided the ASD-associated genes in two subgroups—those that have been identified through a candidate gene approach with knowledge of their function in the cerebellum and their relationship to ASD subsequently confirmed in experimental models, and those identified through unbiased genetic analyses of individuals with ASD, many of which have not yet been characterized extensively and/or not studied in animal models. We also provide recently reported information on non-genetic factors that combine with genetic factors to promote ASD. Together, we hope our review will provide information on recent and significant findings related to the cerebellar underpinnings in ASD.

## 1. Autism Spectrum Disorder (ASD)

ASD is a heterogeneous and behaviorally-defined group of conditions sharing three core phenotypic manifestations-impairment in social communication and interaction, repetitive patterns of behavior, and restricted interests, which typically are displayed early during childhood [1,2,3]. Although not widely appreciated, about 90% of individuals with ASD also have difficulties in sensory processing [4,5,6,7] and about ~30% display intellectual disability [8]. About 15% of young children with ASD have macrocephaly, as a result of accelerated brain growth [9,10]. In contrast to macrocephaly in children, brain size is generally reduced in adults with ASD [11]. Based on current estimates from the CDC’s Autism and Developmental Disabilities Monitoring (ADDM), about 1 in 36 children in the U.S. are diagnosed with ASD [12]. The global prevalence of ASD has increased fourfold over the past three decades. Although much of this increase has been attributed to increased awareness of ASD, increased screening, and better diagnostic tools, a substantial increase in the development of ASD cannot be ruled out. Much of the mechanistic information on the development of ASD has come from rodent models of the disorder. While contributions from these models have been both substantial and critical, it should be noted that there are species-specific anatomical and functional differences between rodents (and even non-human primates) and humans both during brain development and through adulthood that limit the translatability of non-human models to understanding complex brain disorders such as ASD. For example, a unique feature of human brain development is its protracted time course which is crucial to the acquisition of complex circuitry and the higher-order cognitive functions they control [13,14,15]. Recent efforts into understanding the etiology of ASD have utilized human pluripotent stem cell-derived 3D models, such as organoids and assembloids [15,16,17].

Based on clinical criteria, ASD is classified as “syndromic” or “non-syndromic”. Whereas individuals with non-syndromic ASD, also referred to as “idiopathic ASD”, exhibit the core symptoms that define the disorder, syndromic ASD is clinically highly heterogenous and includes other phenotypic abnormalities, such as epilepsy, speech impairment, intellectual disability, and/or dysmorphic features [18,19]. In most cases, syndromic ASD is monogenetic, with the gene mutation causing another neurodevelopmental disorder, the manifestations of which are accompanied by ASD symptoms. Such disorders include Fragile X syndrome, Down syndrome, Asperger’s syndrome, Rett syndrome, and Tuberous Sclerosis Complex (TSC) [20]. It is noteworthy that the affected genes within this category identified so far have disparate biological functions and account for only 1–2% of ASD cases. In contrast, non-syndromic ASD is polygenic with many genes each making minor contributions, and that act in combination with prenatal or perinatal environmental factors [18,19]. While contribution of environmental factors in the pathogenesis of idiopathic ASD is well-accepted, input from genetic factors is much higher and consequently more attention has been placed on identifying the latter. It is noteworthy, however, that there is no known genetic cause for about 80% of individuals with ASD in that the gene(s) involved in most cases of ASD remain to be identified.

While the mechanisms are largely unresolved, there is broad consensus that defective formation, maintenance, and plasticity of synapses play a central role in ASD and other neurodevelopmental disorders [21,22]. Synaptic dysfunction affects neuronal circuitry through a number of effects, chief among which is the disruption of the interplay between excitatory and inhibitory neurotransmission, widely referred to as excitatory/inhibitory (E/I) balance [23,24]. Excitatory (primarily glutaminergic) and inhibitory (primarily GABAergic) synapses are morphologically distinct, contain different protein components, and display different localization. 

The vast majority of effort into understanding the neurobiological underpinnings of ASD has focused on the cerebral cortex and to a lesser extent, the amygdala and hippocampus. Critically, however, a large and growing body of evidence indicates that atypicality in the development and functioning of the cerebellum is more commonly associated with ASD than other brain regions [25,26,27,28,29,30]. In this review we summarize findings that support a key role for the cerebellum in ASD pathogenesis. In particular, we describe many of the genes and signaling pathways the dysfunction of which have been implicated in ASD focusing more on those that are expressed and function in the cerebellum. 

## 2. Cerebellar Structure and Function

**Figure 1:**
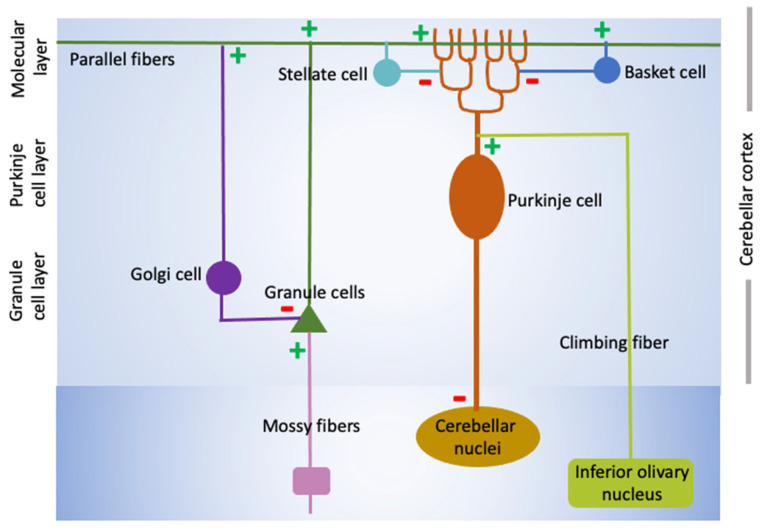
Neuronal circuity in the cerebellum. The mature cerebellar cortex consists of the granule cell layer (GCL), which contains the cell bodies of granule neurons and Golgi cells, the Purkinje cell layer (PCL), which contains the cell bodies of a single monolayer of PCs, and the molecular layer (ML), which contains the dendrites of the PC and granule neurons (parallel fibers), and the basket and stellate cells, two types of GABAergic interneurons. Granule neurons receive excitatory input from the mossy fibers whereas PCs are stimulated by neurons of the inferior olivary neurons. The sole output from the cerebellum is from the PC and through the neurons of the deep cerebellar nuclei. BGs, which localize close to PCs, are not shown in the figure. The + and – symbols indicate excitatory and inhibitory synapses, respectively.

The cerebellum is comprised of three lobes (anterior, posterior, and flocculonodular) which are further subdivided into ten bilateral lobules [31,32]. The outer portion, the cerebellar cortex, is composed of three layers – the internal granule layer (IGL) containing billions of granule neurons, the single-cell Purkinje cell (PC) layer, and a cell sparse molecular layer (ML) in which inhibitory basket and stellate interneurons are located and where synapses involving cell types and granule and PCs exist (Figure 1). The PCs are flanked by Bergmann glial (BG) cells, a specialized type of glial cell present only in the cerebellum, which play a crucial role in the development of the cerebellum and the functioning of PCs through adulthood. Coming into the cerebellar cortex are mossy fibers from brainstem nuclei and the spinal cord, which synapse with provide excitatory input to granule neurons (Figure 1). PCs receive excitatory input from the granule neuron parallel fibers in the molecular layer and from the climbing fibers of the inferior olive. The parallel fibers of granule cell also stimulate basket and stellate interneurons within the molecular layer and Golgi cells, the cell bodies of which reside within the IGL. PCs represent the sole output from the cerebellum receiving inhibitory modulation primarily from stellate and basket cells in the molecular layer (see Figure 1). PCs, which are GABAergic, connect to the deep cerebellar nuclei – the main output nuclei of the cerebellum [31,32]. Unlike neurons within the layers of the cerebral cortex, cerebellar cytoarchitecture within the three layers and ten lobules has long been thought to be relatively uniform and simple. However, recent findings document considerable functional heterogeneity within the same neuronal types across the cerebellum, and with complex region- and cell population-specific transcriptomic and splicing programs underlying its development and functioning [33,34].

Historically considered a motor structure, it is now established that the cerebellum makes functional and anatomical connections to several association areas of the cerebral cortex including the prefrontal cortex and several whole-brain networks that are important for cognition [27,35]. Indeed, like the cerebral cortex, the cerebellum is involved in a variety of cognitive and behavioral functions, including speech and language, emotions, learning and memory, mentalizing, decision making, and reward and social behavior [36,37,38,39,40,41,42,43,44]. Additionally, and through converging connections to sensorimotor cortices, thalamus, and basal ganglia the cerebellum is involved in sensory-motor processing that fine-tunes motor actions [45,46,47]. Defective synaptic signaling within the cerebellum, therefore, will have functional consequences in these other brain regions.

## 3. Cerebellum in Human Studies of Autism

Although several regions of the brain show atypicalities in studies of autism, the cerebellum is strikingly the most consistent site of abnormality. Postmortem studies of autism find reduced PCs in the posterior cerebellum, a region most associated with cognitive processing [48,49,50]. Structural neuroimaging studies in humans also report differences in the cerebellum. Posterior cerebellar regions show reduced gray matter and individual differences in cerebellar gray matter volume are associated with increased symptom severity in core ASD diagnostic criteria (social interaction, language, and repetitive behaviors [51,52]. Reduced cerebellar gray matter has also been associated with language delays in autistic children, one of the first signs of ASD which spurs parents to seek out diagnoses for their child. Finally, cerebellar differences in autistic children and adults have also been documented by functional neuroimaging studies. Posterior cerebellar regions (Crus I/II) implicated in cognition show atypical patterns of connectivity with the cerebral cortex, including regions critical for speech and language, the prefrontal cortex, and other association areas important for social interaction, executive function, and communication [53,54,55]. Atypical functional connections between the cerebral cortex and sensorimotor cerebellar regions (in the anterior lobe and lobule VIII) have also been associated with sensory over-responsivity, a core diagnostic criterion in autism [56].

Crucially, differences in the cerebellum may be an early indicator of long-term outcomes in ASD. For instance, altered cerebro-cerebellar connections in 9-month old infants at risk for autism were associated with greater probability of social communication difficulties years later [57]. This suggests that dysfunction in cerebro-cerebellar circuits might be a useful predictor of social communication disruptions in ASD children even before these behaviors are exhibited.

## 4. Cerebellar Development in ASD

In humans, the cerebellum starts developing from about 4 weeks of gestational age extending into second year of life, with the third trimester of gestation representing a critical period during which there are dynamic changes [58,59].

Cerebellar lesions, damage resulting from tumor-resection, and genetically-caused neurodevelopmental disorders that disrupt cerebellar development, particularly during the critical period, are associated with ASD [26,27,60]. Reduction in cerebellar volume is a common feature both in individuals with ASD and in various mouse genetic models of the disorder [61,62,63]. Specific cerebellar subregions have been identified that are associated with ASD, such as right Crus 1 (RCrus1), which is located in the right posterolateral portion of the cerebellar cortex, and involved in higher-order social and language processing [29]. Compelling evidence that functional connections between the cerebellum and cortex are disrupted in ASD has been described [27]. The cerebellum makes direct connections with the ventral tegmental area (VTA), a brain area critical for the control of social behaviors and reward perception, which in turn makes dopaminergic connections with the prefrontal cortex (PFC) [37]. Optogenetic inhibition of cerebellum-VTA projections in mice abolishes social preference demonstrating the importance of this circuit for normal social behavior. Stimulation of the dentate nucleus (DN) of the cerebellum results in the release of dopamine in the medial prefrontal cortex (mPFC) [64] possibly via the VTA. Altered connectivity between the RCrus1 region of the cerebellum and the PFC has been described in children with ASD and in ASD mice [29]. Together, these findings suggest that reduced or delayed information processing between the cerebellum and regions within the cortex, as well as other brain regions, may manifest as the cognitive, language, motor, and social interactions displayed in ASD. Studies using various genetic models have confirmed the association of cerebellar dysfunction in ASD-like behavior, including sensory defects [65,66,67,68]. It should be noted that while cerebellar underdevelopment or lesions cause cerebellar dysfunction, there are other mechanisms by which dysfunction can occur without obvious changes in cerebellar morphology or volume. For example, exposure to a number of toxins early in life, including mercury, lead, valproic acid, and alcohol [69,70,71,72,73,74,75], as well as prenatal stressors and infections [76,77,78], affect the functioning of cerebellar cells and circuitry without noticeable macro-morphological alterations. 

Of particular significance to ASD pathogenesis are cerebellar PCS, the elaborate dendritic branching of which is the most complex among CNS neurons [79]. The dendrites of PCs are rich in spines which change in form, number, and function in response to stimuli [80,81,82]. Post-mortem examinations of individuals with ASD consistently find a reduction in the number, size, and dendritic arborization of PCs regardless of age or sex [25,61,79,83,84,85,86,87]. In addition to PC numbers, cerebellar weight and size is often reduced in the ASD brain [61,88,89]. Chimeric mice generated using the *Lurcher* mutant (which displays total loss of PCs) and normal mice display repetitive behavior and increased activity, consistent with a causal a role for decreased PC numbers in ASD-related symptoms [90]. Similarly, genetically-induced dysfunction of PCs or their outputs to the deep cerebellar nuclei in mice induces ASD-like behavior, including social interaction deficits, and repetitive and stereotyped behavior [91,92,93]. *Vice versa*, commonly utilized genetic mouse models of ASD that are not constructed by targeting PCs display loss of PCs and cerebellar dysfunction [94]. In addition to reduced PC numbers, disorganization of cerebellar nuclei and a reduction in white matter volume and integrity in the cerebellum has also been described in mouse models of ASD [95,96,97]. In contrast to these changes, neither the density of basket and stellate cells or the number synaptic connections to PCs by these cell types is reduced prior to the loss of PCs [86].

Midgestational exposure of rodents to valproic acid (VPA) recapitulates several of the neurochemical and behavioral features of ASD and is a commonly used non-genetic model of the disorder [98]. Similar to its effect in rodents, exposure to VPA, a drug used to treat epilepsy, during pregnancy increases the risk of ASD [99,100,101,102] strengthening the physiological validity of the experimental model. In VPA-exposed mice, the reduction in PC numbers is unequal across the cerebellar cortex, with only some lobes displaying it [88,89]. Chemogenetic inhibition of PCs in RCrus1 of normal mice generates deficits in social interaction, whereas optogenetic stimulation of this circuit has been described to reduce these symptoms in a mouse genetic model of ASD [29]. This identifies RCrus1 as being one area in which loss of PC could have significant effect on ASD development. Like RCrus1, the cerebellar posterior vermis connects to the mPFC [30]. Stimulation of PCs in the posterior vermis, but not some other cerebellar regions, alleviates repetitive behaviors. This finding suggests that PCs in different parts of the cerebellum control different aspects of ASD [30].

Multiple studies have described gene expression changes in PCs of humans with ASD [103,104,105]. Among the genes that are downregulated is GAD67, which is the major GAD isoform in PCs and in the brain. Given that GAD is the enzyme that catalyzes conversion of glutamate to GABA, a significant reduction in GAD67 expression would lead to reduced GABA production in PCs [106,107,108]. Thus, along with reduced PC numbers, the reduced GAD expression would substantially decrease GABAergic neurotransmission to the deep cerebellar nuclei from the cerebellum to the thalamus and cortical regions with functional consequences. Reduced GAD expression has also been described in the mouse VPA model along with manifestation of ASD-like behaviors, including anxiety, sleep disturbances, and social interaction deficits [89,109,110,111].

An argument by Baizer against a role for the cerebellar dysfunction in ASD has also been presented [112]. Central to this argument is the observation that cerebellar damage does not consistently cause ASD in children. While this is the case, it is undisputable that cerebellar damage increases the risk of developing ASD and is, in fact, regarded as one of the highest risk factors for ASD [113]. Given the highly diverse and heterogenous clinical manifestations and etiology of ASD, it is unlikely that cerebellar damage and dysfunction is the sole contributor to ASD diagnosis. Dysfunction of non-cerebellar brain regions may account for a subset of ASD cases with symptoms that incompletely overlap with cerebellar ASD. Additionally, non-cerebellar areas likely make greater contributions to the systemic symptoms often displayed in ASD, such as circadian rhythm disruption, immune system dysfunction, and gut microbiota alterations [114,115,116]. Finally, even though cerebellar damage greatly increases the risk for ASD, contribution of other genetic and environmental factors may be necessary.

It has also been pointed out that cerebellar damage in adults does not cause the same types of behavioral alterations that ASD children with early-life cerebellar damage exhibit [112]. This is not surprising, however, because early-life damage would have broad impact the development of the cerebellum, its cytoarchitecture, and circuitry within it as well as from it to other brain regions. Being a period of great neuroplasticity, some of the damage resulting from prenatal damage would be alleviated by compensatory mechanisms explaining the milder but broader spectrum of symptoms exhibited by prenatal damage compared with the primarily motor deficits observed in adults.

Another argument made, specifically discounting the role of PCs is that ASD is not observed in all conditions in which there are reduced PC numbers [112]. It deserves mention, however, that reduction in PCs could be due to decreased production during cerebellar development or resulting from increased loss degeneration after these cells are produced in correct numbers. Existing information points to increased death of PCs in ASD, rather than a reduction in their production [117]. In many mutant mouse lines, including those displaying ASD features, PC reduction results from early postnatal degeneration [118,119,120,121] which could impact refinement of neural connectivity in the cerebellum, which would impact functioning of other brain regions that are connected by the cerebellum. In adults, reduction in PC numbers could only result of a loss of pre-formed neurons. Recent findings have shown that subregions within the cerebellar cortex and nuclei have distinct functions [122,123] indicating that the location of PC reduction within the cerebellum would also impact the consequences. Consistent with this, it is known that in different neurodevelopmental disorders, different cerebellar lobules are affected [26,79,124,125]. Additionally, GWAS analyses of different subregions of the cerebellar hemispheres and vermis have described heritable genetic variability across the different anatomical regions of the cerebellum [126,127]. Whether the loss of PCs is accompanied by loss of the associated granule neurons or interneurons could also influence outcome with regard to clinical outcome. Although convincing evidence supports involvement of the cerebellum in ASD, this does not exclude involvement of non-cerebellar areas also, particularly with relation to the systemic symptoms often displayed in ASD, such as circadian rhythm disruption, immune system dysfunction, and gut microbiota alterations [114,115,116].

Strong correlation between circadian rhythm disorders and ASD (as well as other neurodevelopmental disorders) has been documented [116,128]. In humans, and other mammals, the circadian timing system is composed of a central clock in the suprachiasmatic nuclei (SCN) of the hypothalamus and a number of secondary clocks (also referred to as oscillators) in the brain and peripheral organs. The peripheral clocks are synchronized by the central clock through neuronal activity and humoral signals [129,130]. Circadian rhythms both in the central and peripheral clocks are generated by auto-regulatory feedback loops controlled by the CLOCK and BMAL1 proteins, which regulate the transcription of a large number of genes in different organs and cell types to generate oscillations [129,130]. Dysfunction of BMAL1 and some of the clock-controlled genes affect synaptic function and are associated with ASD susceptibility [131]. The cerebellum is one of the many regions outside the central clock that participates in the regulation of circadian rhythm function of the brain [132,133,134]. The cerebellar oscillator has been proposed to reside in PCs [132,135]. Genetic or environmental factors that cause the dysfunction or degeneration of PCs could therefore disrupt the working of the central clock resulting in neurodevelopmental abnormalities, including ASD.

Although it is widely assumed that the underpinnings of ASD lie in neuronal dysfunction, a growing number of studies indicate contributions from abnormalities in astrocytes and microglia. Compelling evidence from both patients and mouse models indicates alterations in the numbers, morphology, and functioning of glial cell types in ASD [136,137,138,139]. By regulating synaptic pruning, microglia play an essential role in the establishment of functional of neuronal circuitry [140,141]. Dysfunction in microglial function resulting in reduced or excessive synaptic pruning disrupts the E/I balance in neuronal circuits affecting brain function and behavior [142,143]. Astrocytes play critical roles in the regulation of a variety of neurodevelopmental processes, including neuronal migration, axon guidance, dendritic morphology, neurotransmitter uptake, and neuroinflammation [144,145,146]. It is now well-documented that astrocytes regulate synaptic development, maturation, and function, and form tripartite synapses with neurons [147,148]. Some of the neurodevelopmental functions of astrocytes involves interaction with microglia [136,149]. Studies of postmortem ASD patients have described increased expression of both astrocyte and microglial markers in the PFC [150]. In the cerebellum however, only an increase in astrocyte markers is found in the ASD along with reduced expression of neuronal markers [150]. Of particular significance to ASD-associated cerebellar dysfunction are BG [151,152,153]. BG play a crucial role in multiple aspects of cerebellar development including neuronal migration, maturation, synapse formation, and regulation of neuronal activity [153,154,155,156]. With regard to PCs, BG regulate the growth and shaping of the dendrites of adjacent PCs [151,155]. In the mature cerebellum, BG processes cover PC synapses, and through their ability to regulate the membrane potential, control the activity of PCs [153,156]. Some evidence suggests that BG regulate the survival of PCs through regulation of glutamate homeostasis [153]. Thus, dysfunction of BG could cause the degeneration and loss of PCs in ASD. Neuroinflammation, oxidative stress and endoplasmic stress in the cerebellum (and other brain regions) are other features described in ASD [25,157,158,159,160,161] and that can result from dysfunction of BGs.

## 5. The Genetic Basis of ASD

ASD is highly heritable-the concordance rate for monozygotic (MZ) twins has been reported to be as high as 90%, and about 31% for dizygotic (DZ) twins [162,163,164,165]. Indeed, along with bipolar disorder, schizophrenia, and ADHD, ASD is among the most heritable of psychiatric disorders. Consequently, much of the research on the molecular underpinnings of ASD has focused largely on genetic factors. While genetic contribution is indisputable, the less than absolute concordance in twin studies indicates that other pre-, peri-, and postnatal environmental factors protect or are necessary for full manifestation of ASD. While the nature of the environmental factors remains poorly understood, maternal lifestyle, pregnancy-related factors including viral infections, birth complications, and parental age have been implicated [166,167,168,169,170].

Consistent with the clinical heterogeneity, different types of inherited or spontaneous genetic mutations, including point-mutations, chromosomal rearrangements, and copy number variants, can cause or contribute to ASD [171,172,173,174]. Another complication in identifying genes associated with ASD is that it can occur with other behavioral disorders such as schizophrenia, bipolar disorder, ADHD, anxiety disorders, epilepsy, obsessive-compulsive disorder, and learning or communication disorders. Results of genetic studies indicate that the genetic risk for ASD can have causal effects on one of aforementioned disorders and *vice versa* [175].

Although the genetic variations that produce the core symptoms of ASD are far from clear, significant progress has been made over the past decade. Various transcriptomic analyses both at the level of brain regions and subregions and from single cells in these locations have been conducted [176,177,178,179,180]. Much of this information has come from the analyses of the cortex, and particularly the PFC. Encouragingly, studies using patients and mouse models have identified a number of common genes the altered expression of which is associated with ASD. But how the heterogenous etiologies and genetic variation in ASD converge on to these common and broadly shared transcriptional changes is currently unclear. In comparison with the cortex, considerably less attention has been placed on ASD-associated genetic variation in the cerebellum although based on existing information, a majority of the genes that have been found cause or increase risk of ASD genes in the cortex and other brain regions are co-expressed in the cerebellum [74,181]. Despite the finding of location-dependent functional differences within the cerebellum, analyses conducted so far using UK Biobank GWAS summary data have not identified specific subregions of the cerebellum displaying significant genetic correlations with ASD at the whole genome-level [126,127]. The lack of genetic correlation may be explained by the canceling out of genetic changes occurring in opposing directions or limited sample size.

In this review, we describe genetic variations that cause or increase the risk of syndromic and/or non-syndromic ASD, focusing, to the extent possible, on the cerebellum. Emphasis has been placed on studies conducted more recently. For this review, we have divided the genes associated with ASD into two subgroups–(A) Those that have been identified primarily through a candidate gene approach and their relationship to ASD then confirmed in experimental models, and (B) Those identified through unbiased genetic analyses of individuals with ASD, many of which have not yet been characterized extensively and/or not studied in animal models.

(A) Candidate gene approach

The candidate gene approach involves identification based on clinical studies, or experimental evidence derived from the analyses of pathophysiological animal models exhibiting ASD-like behavior. Based on the analyses of such candidate genes, primarily using rodent models, a diverse set of cellular abnormalities have been implicated in ASD. Most common among these are abnormal synaptic development, maintenance, functioning, and plasticity [182,183,184]. It is not surprising, therefore, that most of the genes that cause or increase risk of ASD encode for synaptic proteins. We subdivide this section into genes that function primarily at the synapse, and those that have other functions including the development of the cerebellum. Although numerous genes have been implicated in both these categories, focus has been restricted on those that have been implicated in multiple studies conducted by different laboratories and that function in the cerebellum.

A. Proteins regulating synaptic function and neurotransmission

*SH3 and multiple ankyrin repeat domains protein-3 (SHANK3):* The *SHANK3* gene is part of a family of three genes, *SHANK 1–3*, which encode postsynaptic multi-domain scaffolding proteins at glutamatergic synapses in the CNS [185]. As scaffolding proteins, SHANK proteins interact with a large number of postsynaptic proteins and these associations are critical for synapse formation, function, and plasticity [185]. In humans and rodents, all three SHANK proteins are expressed in several brain regions and cell types [185]. Within the human and mouse cerebellum, SHANK2 displays the highest expression in the cerebellum [186,187]. While SHANK1 and 2 are expressed by PCs whereas SHANK3 is expressed in granule neurons [186,187]. The patterns of intracellular localization also differ - SHANK3 mRNA, is localized in the soma of cerebellar cells, whereas SHANK1 and 2 mRNAs are in dendrites [188].

Variations in all three of the SHANK genes are associated with ASD, which totally account for ~2% of ASD cases [189]. Based on mRNA expression and localization analysis, SHANK1 and SHANK2 mRNAs are expressed widely early in the brain, particularly after birth [188]. In contrast, SHANK3 is expressed selectively in the cerebellum and thalamus where expression increases after birth and through adulthood [188]. Within the cerebellum, *Shank1* and *Shank2* mRNA localize selectively to parallel fibers of granule cells. The localization of *Shank* mRNAs to dendrites in the cerebellum suggests roles in synaptic activity-induced alterations through local translation.

Several studies have documented ASD-linked mutations and polymorphisms in the *SHANK1* gene [190,191,192]. ASD core symptoms are recapitulated in SHANK1 knockout mice [193,194,195]. Knock-in mice expressing the R882H mutation, a common SHANK1 mutation in humans with ASD, also display core symptoms of ASD indicating that the mutation impairs function. The development of symptoms is associated with structural changes in the frontal cortex, cerebellum, and hippocampus and a reduction in mGluR1-IP3R1-calcium signaling in these brain regions [196]. Impaired mGluR1 signaling is also displayed in knock-in carrying another common ASD-associated mutation, SHANK1-P1812L [197]. Interestingly, while mice hemizygous for the mutant allele display core ASD symptoms, homozygous mutant mice displayed long-tern memory impairment but not ASD behaviors [197].

Loss-of-function mutations in the *SHANK2* gene are a particularly penetrant cause of ASD with cerebellum-regulated motor impairment [189]. PC-specific deletion of SHANK2 in mice results in their dysfunction and in an ASD-like phenotype [198]. In mouse models of both idiopathic and syndromic ASD, GABA-A receptor density is reduced in the cerebellum which disrupts E/I balance [199]. Interestingly GABA-A receptor density is elevated in the ASD hippocampus in SHANK2-deficient mice suggesting brain-region-specific perturbations. Together, these and other studies indicate that dysfunction of both excitatory and inhibitory neurotransmission contribute to ASD.

Most research of the involvement of SHANKs in ASD has been on SHANK3 [200,201,202]. SHANK3 gene haploinsufficiency is the major cause of Phelan-McDermid syndrome (PMDS), a syndromic form of ASD [203,204]. Other loss-of-function mutations of SHANK3, which include large deletions and insertions, point mutations, and splicing mutations, have been linked to non-syndromic ASDs [189,205,206,207]. SHANK3-deficient mice and rats display disrupted E/I balance, and alterations in dendritic and spine morphology in multiple brain regions, including the cerebellum. Furthermore, the mutant animals display ASD-like behaviors including repetitive grooming and impaired social interaction [200,208,209,210]. Loss of SHANK3 function also produces ASD-like behavior in other species, including zebrafish [211], dogs [212], and macaques [213]. Results of a recent study described deregulated glutaminergic receptors at granule neuron - mossy fiber synapses in SHANK3 mutant mice, which correlated with behavioral alterations [214].

*Neuroligins (NLGNs):* One of the many proteins that interact with SHANK proteins in the postsynaptic terminal are the neuroligins (NLGNs), a family of four transmembrane cell adhesion proteins, (NLGN1-4), that regulate synapse formation, organization, and function [215]. Besides associating with postsynaptic proteins though their intracellular domains, the extracellular domain of NLGNs bind to presynaptic transmembrane neurexin proteins forming bridges across synapses. Whereas the other NLGNs act at both excitatory and inhibitory synapses, NLGN2 acts exclusively at inhibitory synapses [216]. Mutations in all for NLGN-encoding genes display a high penetrance linkage to ASD [217]. The mutations are generally de novo and occur in the germline [217]. Among the NLGNs, genes for NLGN-3 and NLGN-4 are on the X-chromosome [215].

Each NLGN plays an essential role in synapse maintenance as RNAi-mediated knockdown of each of them results in extensive loss of both excitatory and inhibitory synapses [218]. Analyses of the localization of the four NLGN proteins has revealed that NLGN1 is specifically present in excitatory neurons, NLGN2 in inhibitory neurons, and NLGN4 in glycinergic neurons [218,219,220,221]. In contrast, NLGN3 is expressed in both excitatory and inhibitory neurons. While the intracellular region of the NLGNs associate with postsynaptic proteins, including SHANK proteins, the extracellular region interacts with neurexins (NXNs), which are transmembrane presynaptic proteins. The NLGN-NXN interaction is necessary for synapse formation and maintenance [222,223,224]. Within the postsynaptic cell, NLGNs also play a key role in localizing neurotransmitter receptors and channels [225]. Different NLGN mRNAs can be co-expressed in the same neurons where they are localized to distinct types of synapses [226,227].

All four NLGNs are expressed in the cerebellum [226]. Selective deletion of NLGNs in cerebellar PCs either singly or in combination reveals that NLGNs have both shared and distinct roles in regulating synaptic transmission [228,229,230]. Ablation of all NLGNs in PCs results in the reduction in climbing fiber synapses [228]. NLGNs also localize within cerebellar astrocytes with about 40% of the total NLGN expressed in BG cells, which as described above, is the predominant astrocyte in the cerebellum.

In the cerebellum, NLGN1 localizes mostly to synapses between parallel fibers, processes of interneurons in the ML, and synapses of mossy fibers and granule cell dendrites where it colocalizes with PSD-95, which marks excitatory postsynaptic sites [231]. In contrast, localization of NLGN1 is low in PC dendrites and soma. NLGN2 localization in the cerebellum is restricted to inhibitory synapses and plays a critical role in regulating climbing fiber synapse numbers [228]. NLGN3 is localized predominantly in the cell body of BG cells with much lower presence in the processes [230]. Little is known about the localization pattern or function of NLGN4 in the cerebellum.

*Nlgn1* and *Nlgn2* knockout mice display ASD-like behaviors [232,233]. In the Fragile-X mouse model of ASD, the expression of NLGN1 is decreased in the brain [234]. Normalizing NLGN1 expression alleviates impairment of social behavior in mutant mice, but not the deficit in learning and memory suggesting that the reduced level of NLGN1 is a key contributor to at least some ASD-like behavioral impairment [234].

In the context of ASD, most work has been performed on NLGN-3. In the cerebellum, NLGN-3 is expressed both in PCs and granule neurons. Knock-in mice with an ASD-associated mutation in NLGN-3, R451C, a mutation first identified in individuals with ASD [235,236], display increased cortical inhibitory synaptic strength along with impaired social interaction [62,237,238,239]. Another study that compared the effect of the R451C mutation in different brain regions described increased excitatory synaptic transmission in the hippocampus but increased inhibitory transmission in the somatosensory cortex indicating that the same mutation induces region-specific changes in synaptic function [239]. In the cerebellum of NLGN-3-R451C mice, the expression of NLGN3 is reduced by ~90% suggesting that the mutation renders NLGN3 unstable [240]. Furthermore, the normal postnatal pruning of supernumerary climbing factor synapses with PCS is impaired, leading to abnormal E/I balance in PCs [240]. Abnormal synapse elimination is thought to contribute to ASD and other neurodevelopmental disorders [241]. In contrast to mice with the R451C mutation, NLGN-3 global knockout mice do not display an ASD phenotype suggesting a gain-of-function effect of the R451C mutation [242].

NLGN-3 is also expressed in cerebellar BG cells although its absence in these cells in mice does not affect morphology, synaptic number, or synaptic function [230]. However, BG-specific NLGN-3 deletion does alter gene expression in other cerebellar cell types, including PCs, granule neurons, and oligodendrocytes, as well as in the cortex [230]. The contribution of BG-initiated changes in gene expression in the cerebellum and cortex to ASD associated with NLGN3 deficiency remains to be clarified. Somewhat puzzlingly, NLGN-3-R451C mutation in mice of another genetic background than the one used in the aforementioned studies display minor behavioral issues but not ASD-like behaviors [243] indicating that ASD-like behavioral outcome caused by the R451C mutation is dependent on genetic factors.

Aberrant cerebellar - cortical communication is likely to be an important contributor to ASD [27,29,60]. In addition to alterations within the cerebellum, inhibitory output from the deep cerebellar nuclei to the thalamus, midbrain and brainstem is reduced in ASD-NLGN-3 mice leading to impaired social interaction [93]. Of these different pathways, chemogenetic inhibition of the pathway connecting to the zona incerta (ZI), a subthalamic region, rescues social impairment in ASD-NLGN3 mice [93]. Specific deletion of NLGN3 in cerebellar astrocytes, which normally express it highly, results in modest transcriptional changes among multiple cell types, but not in synapse number, synaptic transmission, or morphology of the astrocytes [230]. In contrast, deletion of NLGN2 in astrocytes, albeit of the cortex, affects morphology and synaptic transmission in addition to gene expression changes [244]. The consequences of NLGN2 in cerebellar astrocytes or BG cells have not been studied.

Mutations in the X-linked *NLGN4* gene represent one of the most common monogenic causes associated with ASD with more than 50 mutations identified in humans [3,245,246]. NLGN4 is widely expressed in the brain localizing primarily to inhibitory glycinergic synapses where it is required for the organization and functioning of these synapses [247,248,249]. Localization to excitatory synapses has also been described [250,251]. Mutations of NLGN4 could thus contribute to ASD by affecting synaptic transmission and impairment of E/I balance. Mice lacking NLGN4 display impaired social interaction, repetitive behavior, and other ASD behaviors [248,252]. Knock-in mice heterozygous for an ASD-associated NGNL4 mutation, P89L, also display abnormal social behavior suggesting that the mutation reduces function. The P89L mutation affects the cellular localization of NLGN4 and impairs dendritic spine formation [253]. Another ASD-associated genetic alteration in NLGN4 is missense mutation at an arginine conserved in all four NLGN members, R704C. In NLGN4 the R470C mutation elevates AMPA-receptor-mediated synaptic responses. However, when the same mutation is created in NLGN3, it enhances AMPA-receptor internalization resulting in reduced postsynaptic AMPA-receptor at the synapse [254]. Therefore, while both NLGN3 and NLGN4 affect the AMPA-receptor they have different effects on it and consequently on AMPA-receptor-mediated synaptic function. In contrast, another ASD-associated mutation, R87W, blocks the transport of NLGN4 to the cell surface thus blocking its actions on synaptic formation and function [255]. Similarly, an ASD-linked R101Q mutation impairs maturation of NLGN4 resulting in its retention in the ER and Golgi reducing its transport to the membrane [251].

*Classical neurexins (NRXNs):* NRXNs consist of a superfamily of presynaptic cell adhesion proteins that are subdivided into two groups - classical NRXNs encoded by three genes (NXRN1-3) and a large number of related proteins belonging to the contactin-associated protein (CASPR) subfamily. The *NXRN 1–3* genes produce a multitude of transcripts through alternative splicing and promoter usage that are expressed in a cell-specific manner [256,257]. Besides interacting with NLGNs, NRXNs associate with a variety of postsynaptic proteins [220,257]. Through these interactions, NRXNs regulate a number of synaptic properties, including assembly of the synapse, the organization of the presynaptic release machinery, and the regulation of E/I balance [258]. Mutations in all three NRXNs are linked to ASD [258,259,260]. Interestingly, the three NRXNs display distinct patterns of expression in the developing human cortex [261] suggesting that their dysfunction contributes to ASD through distinct mechanisms.

In the cerebellum NRXNs connect to postsynaptic proteins through interaction with cerebellins (CLBNs), a family of four secreted adaptor proteins [262]. Association of NRXNs with CLBNs, particularly CLBN1, is instrumental for the development of cerebellar parallel-fiber synapses [262].

Genetic variants in all three *NRXN* genes are associated with ASD [257,263,264]. In the context of ASD, most attention has been placed on NRXN1 [265,266,267]. Mice lacking NRXN1 display electrophysiological abnormalities and exhibit ASD-like behavior [268,269,270] indicating a loss-of-function mechanism in ASD. Similarly, NRXN2 knockout mice display synaptic abnormalities and ASD-like behavior [258,271]. Analysis of different *Nrxn3* knockout mouse lines and brain regions reveal that NRXNs has distinct roles in different brain regions and on excitatory versus inhibitory synapses [272]. Deletion of some isoforms of NRXN3 impairs dendritic synapses but not somal or axonal synapses [273].

*CNTNAP2: CNTNAP2* is the gene that encodes contactin-associated protein 2 (CASPR2), a transmembrane protein belonging to the CASPR subfamily of the NXRN super-family. CASPR2 is widely expressed in the CNS [274,275]. Besides functioning as a cell adhesion molecule, CASPR2 is involved in localizing K+ channels at Nodes of Ranvier, regulating myelination, and in cell-cell interactions [275,276]. CNTNAP2 mutations [277,278,279] and expression-reducing variants of the CNTNAP2 promoter region [280] are associated with ASD in humans. CNTNAP2-deficient mice also display synaptic dysfunction and ASD-like behaviors [281] supporting a loss-of-function mechanism.

CNTNAP2 is also widely expressed in the cerebellum [282]. Individuals with ASD-related mutations in the CNTNAP2 gene display reduced gray matter volume in the cerebellum [283], a consistent feature in humans with ASD. Similarly, mice lacking CNTNAP2 display reduced cerebellar volume [284]. CASPR2 is abundant at cerebellar synapses and CNTNAP2 knockout mice display disrupted dendrite development of PCs and impaired motor coordination [285]. Another study described altered morphology of PCs in cerebellar Crus I/II region of mutant mice and impaired electrical responses to somatosensory stimulation, indicating abnormal sensory processing by the cerebellum [67]. CNTPNAP-deficient mice have been reported to develop cerebellar heterotopias (as do a subset of ASD patients) although one study has attributed this to the genetic background of C57BL/6 mice, which develop spontaneous cortical and cerebellar heterotopias [286].

Much more is known about the role of CNTNAP2 in the cortex. During cortical development, CNTNAP2 is highly expressed in early-born excitatory cortical neurons. Loss of CNTNAP2 disrupts the excitatory neuron differentiation as well as overall neural circuit assembly in the developing cortex [287,288]. Other functions of CNTNAP2 in the cortex include the regulation of neuronal migration, glutamate receptor organization, and morphology of dendritic arborization and synaptic spines [288,289].

*Cadherins:* Cadherins are a large family of about 100 cell adhesion proteins, which in the nervous system are involved in the formation of neural circuitry, organization of synapses, and regulation of synaptic plasticity [290,291,292,293]. Besides mediating cell-cell contact, cadherins also regulate intracellular signaling [292,294,295]. The large family of cadherin proteins is subdivided into multiple sub-families, including Type I cadherins, which are widely distributed, Type II cadherins, which that are expressed in specific brain regions and subcellular compartments, protocadherins, and atypical cadherins [296]. In the cerebellum, many cadherins are expressed at the earliest stage of development and where their function is required for proper cerebellar development [294]. One function of the cadherins in the developing cerebellum is to regulate migration of PCs to parasagittal regions and ensure proper connectivity [294,297,298]. Unbiased genetic studies have identified mutations in several cadherins in neurodevelopmental disorders, including ASD [299,300,301]. Indeed, copy number variations in multiple Type-II cadherins have been linked to ASD [302]. A study focusing on two ASD-associated Type II cadherins, CDH9 and CDH11 within the cerebellum, described high expression in non-overlapping populations of PCs during early development but a decrease in as cerebellar development proceeded [303]. In rodents, CDH11 expression is largely restricted to dorsal lobules of the vermis and the lateral hemisphere area equivalent to the Crus I and Crus II areas in the human cerebellum [303]. The high CDH11 expression correlates with the expression of the calcium-binding protein, calbindin, and is associated with a delayed maturation of PCs [303]. Together, these results suggest that the two ASD-linked cadherins function to regulate the development of the cerebellum and the circuitry within it. Although elevated expression affects PC development, deletion of a portion of the CDH11 locus has been described in idiopathic ASD [304]. Similarly, organoids generated from ASD patient-derived iPSCs display reduced CDH11 expression [305]. Another study using cultured CDH11-deficient hippocampal neurons described increased expression of postsynaptic density (PSD)-95 and NLFG1 which accompanied changes in dendritic morphology and electrical properties [305]. The somewhat counterintuitive elevation in PSD-95 and NLGN1 expression were suggested to represent a compensatory effect. In sum, both increase and decrease in CDH11 function could contribute to defective maturation and function of neurons that contribute to ASD. In addition to CDH9 and CDH11, mutations in CDH13, a cadherin expressed specifically within Golgi cells of the cerebellum, has been linked to ASD [302,306]. Mice lacking CDH13 in Golgi cells exhibit reduced display deficits in cognitive performance and social behavior, but not in motor function [307]. Mice lacking CDH13 display deficits in adaptation to early-life stress [308].

Besides Type-1 and Type-II cadherins, some members of the atypical cadherin subfamily have been linked to ASD. Among these is FAT1 (FAT atypical cadherin-1), a protein that is expressed highest in the postnatal cerebellum, where it is localized to granule neurons and Golgi cells in the IGL and inhibitory interneurons in the molecular layer [305,309,310]. The expression of FAT1 is reduced in iPSC-derived neural cells from individuals with ASD [311]. Another atypical cadherin CELSR3, which is necessary for proper brain development [312,313], is expressed highly in PCs. Mice with elective deletion of Celsr3 in PCs display some ASD-like abnormalities, including reduced dendritic arborization and synaptic plasticity, and motor impairment [314].

*CUB and sushi multiple domains 3 (CSMD3):* Mutations in CSDM3, a member of a family of three proteins (CSDM1-3), have been linked to ASD [315,316]. CSMD3 is a large oligomeric transmembrane protein, expressed primarily in the fetal and adult brain [315]. In the postnatal mouse hippocampus, CSDM3 localizes to apical dendrites [317]. Elevating CSDM3 expression promotes dendritic branching in cultured hippocampal neurons, an activity that requires its extracellular domain suggesting that interaction with another protein is involved [317]. In fact, CSDM3 has been proposed to act as a coreceptor with some other as yet unidentified protein [317]. Mice lacking CSMD3 display core ASD symptoms and motor deficits which has been attributed to cerebellar dysfunction [318]. Specifically, CSMD3-deficient mice display abnormal PC morphology in Right Crus I/Crus II lobules along with E/I imbalance in PC synapses within these cerebellar lobules. Besides its effects in the cerebellum, CSMD3 deficiency impairs neurogenesis and synaptogenesis in the cortex perturbing functional neural networks [319].

B. Proteins involved in cerebellar development and other cellular functions

*Engrailed-2 (EN-2-):* EN-2 is a homeobox transcription factor that is expressed at high levels in the cerebellum and hindbrain and is necessary for pattern formation and connectivity of the cerebellum during development. *En-2* knockout mice display reduced cerebellar size, altered foliation of lobes, reduced number of PCs, and abnormal mossy fibers [320,321,322,323,324]. The number of astrocytes and microglia are increased in the cerebellum of *En-2* knockout mice [325]. More recent studies describe roles of EN-2 in regulating neuronal differentiation, diversity and survival of cerebellar neurons [326]. Expressing EN-2 in mice beyond the time that it is normally downregulated results in a delay in the onset of PC differentiation suggesting that EN-2 controls the timing of PC differentiation [327].

Genetic studies conducted by multiple groups have identified *EN-2* as an ASD susceptibility gene [328,329,330,331]. Susceptibility in humans is conferred by intronic SNPs that increase transcription of the *EN-2* gene [332,333]. Additionally, elevation of En-2 gene transcription in the ASD cerebellum results from increased activating histone H3K27 trimethylation [334] or by reduced repressive binding of MeCP2 (Methyl CpG-binding protein) to methylated regions in the 5’ region of the En-2 gene promoter [335]. In view of the finding that EN-2 negatively regulates PC differentiation [327], the increased EN-2 expression in the ASD cerebellum could interfere with normal PC maturation and function, thereby resulting in cerebellar dysfunction. Surprising in view of the elevated En-2 expression humans with ASD, mice lacking EN-2 also display ASD-like neurochemical alterations and behavioral phenotypes, including cognitive impairment and deficits in social interaction [336,337]. Transcriptomic analysis of the cerebellum of mice lacking EN-2 reveal increased expression of genes regulating immune function with reduced levels of pro-inflammatory molecules and chemokines further establishing relationship between immune dysfunction and cerebellar deficits in ASD and a role for EN-2 in this imbalance [338]. 

*Tuberous sclerosis complex (TSC) proteins:* Loss-of-function mutations of the *TSC1* or *TSC2* gene in humans causes a multiorgan disorder called TSC, which is characterized by non-malignant tumors in the brain (and several other organs), white matter abnormalities, intellectual disability, and epilepsy [339,340]. A majority of TSC patients also display ASD behavior [341,342,343] with *TSC2* gene mutations causing more severe symptoms than *TSC1* mutations [344]. Mice in which TSC1 is specifically deleted in PCs exhibit core ASD features, including social interaction deficits, repetitive behavior and vocalizations, and cognitive deficits [345]. Similarly, mice lacking TSC2 display progressive loss of PCs, cerebellar dysfunction, and ASD behavior [346]. Interestingly, TSC2-deficient mice that are seizure- and lesion-free do not display cognitive impairment [347] supporting the possibility suggested by some that cognitive deficits in individuals with ASD might be caused or potentiated by seizure-induced damage [348].

**Figure 2:**
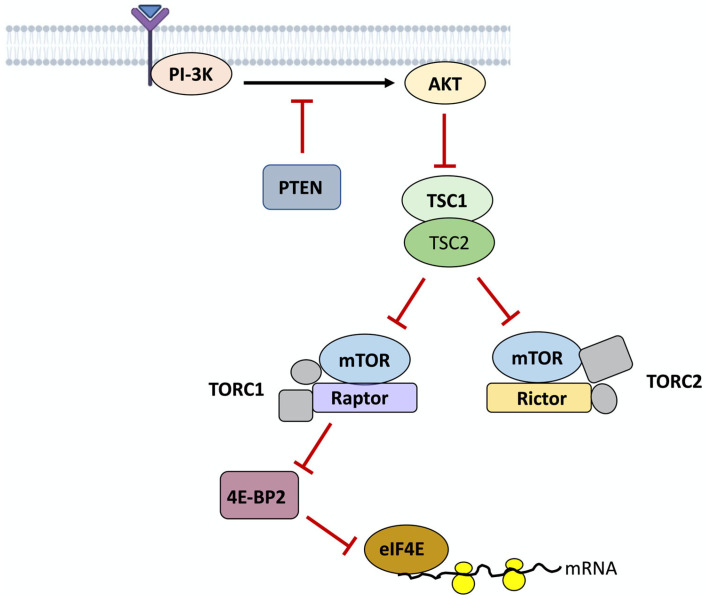
The AKT-mTORC signaling pathway. Through phosphorylation of membrane-associated phosphoinositides, activation of the lipid kinase PI-3 Kinase following the binding of growth and neurotrophic factors to their receptors leads the activation of AKT, a cell survival-promoting protein kinase. AKT inhibits the TSC1/TSC2 protein complex which inhibits mTOR. mTOR, along with RAPTOR and RICTOR, is part of multiprotein complexes, TORC1 and TORC2, respectively. Through phosphorylation, mTORC1 inhibits 4E-BP, which normally binds the translation initiation factor eIF4E inhibiting it. Phosphorylation by 4E-BP2 causes the disassociation of 4E-BP2 from eIF4E promoting ribosome recruitment and protein translation. AKT-mTORC1 signaling can be inhibited by PTEN, a lipid phosphatase by reversing PI-3K-mediated phosphorylation of phosphoinositides.

TSC1 and TSC2 negatively regulate mammalian target of rapamycin (mTOR), which forms two distinct types of protein complexes, mTOR complex-1 (mTORC1) and mTORC2 which differ in composition, intracellular localization, regulation, and substrates [349,350,351] (Figure 2). With regard to differences in composition, TORC1 contains the scaffolding protein Raptor, which is essential for its activity, whereas TORC2 contains Rictor, a protein required for its activity (Figure 2). Additionally, mTORC1 is substantially more sensitive to rapamycin than mTORC2. A major function of TORC1 is enhancing of protein synthesis, with other functions including negatively regulating autophagy, while the major function of TORC2 activates kinases that regulate cell survival and metabolism, including lipogenesis and glucose transport [352,353].

In neurons, mTORC1 regulates dendritic morphology and synaptic density [354,355]. Abnormal increase in mTORC1 activity (because of reduced TSC1/TSC2 inhibition) along with abnormal dendritic morphology have been described in patients with ASD with the severity of symptoms correlating with the level of increase [355,356,357]. In mice, increased mTORC1 signaling leads to extra-numerary striatal - cortical excitatory synapses. The surplus synaptic connection in TSC2-deficient mice has been attributed to reduced autophagy and synaptic pruning resulting from overactivity of mTORC1 [358,359]. In a TSC2-deficient rat model of ASD, the cerebellar vermis shows enlarged white matter, a thickened molecular layer, and demyelination of the central tract of the vermis [360]. Furthermore, and as observed in mutant mice and ASD patients, ASD-like rats display reduced PC numbers and increased numbers of astrocytes and microglia. However, BG numbers and morphology are unchanged. Pharmacological inhibition of mTORC1 stimulates autophagy, reduces the synaptic hyperactivity, and ameliorates ASD behaviors in mice and restores normal neuronal connectivity and activity in iPSC-derived neurons from humans with TSC2 mutations [358,359,361,362]. Based on such results, mTORC1 has been considered as a target for the treatment of ASD and clinical trials are underway to test the efficacy to mTORC1 inhibitors [356,363]. 

Knocking out TORC1 activity specifically in PCs through deletion of the *Raptor* gene results in social interaction impairment and progressive loss of PCs [364]. In contrast, PC-specific deletion of *Rictor*, which eliminates mTORC2 activity, results in motor coordination and the gait alterations and disruption of climbing fiber synapses [364]. These results demonstrate that both mTORC1 or mTORC2 play important roles in cerebellar development and that deletion of either produces non-overlapping ASD-like phenotypes. Furthermore, these results show that either loss or gain of mTOR activity produces ASD-like pathologies pointing to the importance of careful regulation of mTOR activity for proper cerebellar development.

An important downstream target of mTORC1 is eIF4E, a key component of a multiprotein complex required for translation of capped mRNA [365,366] (Figure 2). Within the complex, binding of eIF4E to the mRNA cap leads to the recruitment of the ribosomal subunits for translation to initiate. The formation of the eIF4E complex depends on the phosphorylation of eIF4E-BP2, a binding protein that interacts with eIF4E inhibiting it. Phosphorylation of eIF4E-BP2 by mTORC1 causes its disassociation from eIF4E resulting in eIF4E-mediated complex formation and mRNA translation and thus in increased protein synthesis (Figure 2). Thus, mTORC1 promotes cap-depended protein synthesis by reducing the inhibitory action of eiF4E.

Several lines of evidence point to elevated eIF4E function as a key contributor of ASD. Deletion of the gene encoding 4E-BP2 or overexpression of eIF4E in mice increases protein synthesis in the brain and leads to ASD-like behaviors [367,368,369]. 4E-BP2-lacking mice also display a disruption of the E/I balance in the hippocampus [367,370]. Surprisingly, cell type-specific deletion of 4E-BP2 in GABAergic interneurons of mice produces ASD-like behaviors, whereas deletion in forebrain glutaminergic excitatory neurons or astrocytes do not [371]. Mice in which 4E-BP2 is specifically deleted in PCs display a reduced number of PCs, aberration in neuronal activity, and impaired motor and spatial learning [372]. However, these mice do not display social interaction or repetitive behavior suggesting that in the cerebellum, ASD-like impairment of social and repetitive behavior is controlled by a mechanism that is different from the control of motor and spatial memory [372].

Among the mRNAs that display increased translation in the hippocampus of *4Ebp2* knockout mice are *Nlgn1-4* mRNAs [367]. Knockdown of NLGN1 but not NLGN2 restores E/I balance, restores normal protein levels, and reverses ASD-like behavior in the 4E-BP2 knockout mice [367]. As observed in 4E-BP2 knockout mice, pharmacological inhibition of NLGN1 or its knockdown reverses ASD-like behaviors in TSC2-deficient mice without a reduction in mTORC1 activity [373], confirming that hyperactivated mTORC1-mediated ASD-like phenotype results from eIF4E-induced NLGN1 synthesis. Interestingly, recent evidence suggests that the ASD-associated deregulation of eIf4E function occurs in microglia [374]. Overexpression of eIF4E in microglia, but not neurons or astrocytes, leads to ASD-like behaviors [374]. Interestingly, although microglial eIF4E overexpression elevates translation in both sexes, dysfunction of microglia occurs only in male mice [374]. 

*Phosphatase and tensin homolog (PTEN):* PTEN is a tumor suppressor that negatively regulates cell division and survival, acting as a lipid phosphate to inhibit the PI-3 Kinase-AKT and mTOR pathways [375]. PTEN is required for the normal development of the cerebellum, regulating cerebellar cytoarchitecture and migration of neurons and glial cells [376]. Not unexpectedly, germline PTEN mutations in humans results in structural and functional abnormalities in the cerebellum [377]. Astroglial deletion of PTEN results in disorganization of cytoarchitecture predominantly in the cerebellum and hippocampus [378].

Initial findings of reduced PTEN levels in ASD patients with macrocephaly [379] led to genetic analyses that revealed ASD-associated germ-line mutations in the *PTEN* gene [380,381,382,383,384]. Germline PTEN mutations in humans with ASD are associated with extreme macrocephaly [380,385]. While only responsible for a relatively small proportion of ASD cases, most individuals with loss-of-function PTEN mutations and macrocephaly present with ASD (those that do are designated as ASD-PTEN). PTEN-deficiency in the brain results in hypertrophy of the soma and abnormal migration, increased dendritic elaboration, dentritic overgrowth, and higher spine density, which together result in neuronal hyperexcitability [386,387,388,389,390,391,392,393]. Mice in which PTEN is selectively deleted in PCs display structural and functional PC abnormalities and exhibit ASD-like behavior [394]. As described above, PTEN inhibits Akt activity, which inhibits TSC1/TSC2, which inhibits mTORC1/mTORC2. Not unexpectedly, therefore, mTORC1 activity is elevated in ASD-PTEN patients and in PTEN-deficient mice and chronic administration of rapamycin (which also inhibits mTORC2 upon prolonged exposure) ameliorates ASD-like behavior and brain abnormalities in PTEN-deficient mice [386,388,390,395].

It is noteworthy that although much of the effects of PTEN are mediated through its actions at the plasma membrane, where it inhibits PI-3 Kinase-AKT and mTOR signaling, PTEN also localizes to the nucleus. Selective reduction in nuclear PTEN in mice results in macrocephaly at birth, reduced neuronal soma size in the cortex, cerebellum, and hippocampus, and enhanced seizure susceptibility [396]. The contribution of nuclear PTEN to ASD remains uninvestigated. 

*Brain and Muscle ARNT-Like 1 (BMAL1):* ASD is often associated with circadian clock perturbations, which result in sleep disturbances [397,398]. Based on a number of studies indicate that 50–80% of developing children with ASD have sleep disturbances [131,398,399]. In the VPA-induced ASD mouse model, circadian behavior and expression of circadian clock-regulatory genes is altered [400]. A key protein in the regulation of the circadian clock is BMALl, transcription factor which heteromerizes with the CLOCK transcription factor to activate transcription of the *PER* and *CRY* genes. Once produced PER and CRY form a complex that inhibits the transcriptional actions of BMAL1-CLOCK resulting in oscillatory transcriptional- feedback loops [401]. Both *Bmal1*-null and -haplosufficient mice develop core behavioral deficits of ASD including social impairments, repetitive behaviors, and learning disabilities [402,403]. BMAL1-deficient mice also display aberrant cell density and immature morphology of dendritic spines. Moreover, the mutant mice display abnormal cell density in the cerebellum along with immature morphology, and electrophysiological properties of PCs [402]. Deletion of BMAL1 only in PCs recapitulates the ASD-like phenotype, displayed by BMAL1-null mice [402] underscoring the importance of PC dysfunction in ASD.

Besides being a critical component of circadian rhythm regulatory machinery, BMAL1 has other functions including the regulation of mRNA translation [404], a process that is under the control of TORC1 activity (as described above). Interestingly, mTORC1 and eIF4E activity, deregulation of which is associated with ASD, is increased in the BMAL1-knockout cerebellum [402]. Additionally, the transcriptional profile in the cerebellum is altered with changes in the expression of several ASD-associated genes. Pharmacological inhibition of mTORC1 signaling reverses the ASD-like behavior in BMAL1-deficient mice, indicating that the impairments resulting from BMAL1 deletion are mediated by hyperactivation of mTORC1 [402].

While BMAL1 deficiency leads to an hyperactivation of mTORC1, mTORC1 prevents the degradation of BMAL1 through its phosphorylation by S6 kinase, an effector target of mTORC1. Consequently, in BMAL1 protein expression is increased in TSC2-deficient cells and mice, an experimental model of ASD [405]. Treatment with an mTOCR1 inhibitor reverses the increased BMAL1 expression whereas reducing the level of BMAL in mice normalizes the circadian phenotype in TSC2-deficient mice. These findings indicate that the ASD-promoting effect of BMAL1 deficiency results from mTORC1 hyperactivation [405].

It is noteworthy that besides regulating the circadian clock and protein translation, BMAL1 regulates mitochondrial fission and mitophagy [406], autophagy [407], neuroinflammation [408], synaptic pruning [409], and myelination [410], all processes that are dysregulated in ASD. Therefore, BMAL1-deficiency can contribute to ASD by TORC1 dependent and independent mechanisms.

*Retinoic acid-related Orphan Receptor alpha (RORα):* RORα is best known as a nuclear receptor that is widely-expressed in the brain and that is activated through binding by retinoic acid (RA), a powerful signaling molecule produced through the oxidation of retinal (vitamin A) [411,412]. RA plays a variety of critical roles during CNS development, including the regulation of stem cell production, neuronal differentiation, and morphogenesis of brain structures [413,414]. RA also regulates synaptic plasticity, E/I balance, and other important processes in the adult brain [415].

Nuclear RA-bound RORα exerts its biological effects by binding to genomic DNA at ROR response elements (RORE) located within the promoters of a large number of genes [416]. More importantly, RORα binds to the promoters of over 400 genes that have been implicated in ASD [412,417,418,419]. In humans, mutations in the RORα gene can cause either ASD or cerebellar ataxia depending on whether the mutation produces loss-of-function or a toxic gain-of-function, respectively [420].

RORα plays a key role at different stages of cerebellar development and through adulthood [412]. In the cerebellum, RORα is most highly expressed in PCs and is required for the development and survival of these neurons [421,422,423,424]. Mice lacking RORα display defective interactions between PCs and climbing fibers, atrophy of the soma and dendrites of PCs followed by their death resulting in cerebellar dysfunction [422,425,426]. In the prenatal VPA-exposure rat model of autism, reduced PC numbers and arborization are accompanied by reduced level of RA and decreased expression of RORα. Administration of RA to VPA-exposed rats normalizes expression of RORα, PC number and dendritic arborization, and improves motor function [427]. Although exactly how reduced function of RORα contributes to ASD is not known, given the large number of target genes with varied functions, it is possible that deregulation contributes to several of the diverse symptoms in ASD. One target gene that could be particularly important is RA-induced 1 (RAI1), a protein that is expressed at high levels in the cerebellum and in PCs, and that is involved in cognitive and motor function [428,429]. RA administration stimulates expression of RAI1, while decreased function of RAI1 has been described to contribute to multiple neurodevelopmental disorders [429,430,431].

Besides neurons, RORα is expressed in astrocytes where it acts to suppress inflammation and promote neuronal survival [432]. It also expressed in microglia, a cell type that changes morphology during sleep [433]. Reduced RORα in microglia function leads to reduced BMAL1 expression and disrupts the microglial clock system, which contributes to sleep disruption but also deficits in social interaction and cognitive impairment [433]. Microglial RORα also suppresses neuroinflammation [433], an alteration linked to ASD.

*E3 ubiquitin ligase 3A (UBE3A):* UBE3A is a protein which functions both as an E3 ubiquitin ligase and a transcriptional regulator for steroid hormone receptors, which functions to regulate synaptic function and plasticity in the brain. It is expressed widely in the brain, with high expression in the cerebellum where it is expressed in PCs and Golgi cells [434,435,436]. In healthy individuals, the region encompassing the UBE3A gene is silenced by imprinting in the paternal copy of the chromosome resulting in only the maternal copy of the gene being expressed. Mutations or imprinting errors that disrupt expression of the maternal allele results in reduction or complete loss of *UBE3A* gene expression, causing Angelman syndrome (AS), a disorder characterized by ASD features [437,438]. In AS mice, maternal allele imprinting caused marked reduction of UBE3A in the cerebellum and hippocampus, whereas loss in other brain regions was low to moderate [434,439]. Within the cerebellum of AS mice, a substantial loss of UBE3A occurs in a subset of GABAergic Golgi interneurons in the granule cell layer [436,440]. Duplication, triplication, or gain-of-function mutations in the *UBE3A* gene are also associated with ASD behavioral features indicating that the expression and activity of UBE3A must be tightly regulated during brain development. The proteins degraded through the activity E3 ligase activity of UBE3A remain largely unidentified.

Elevated UBE3A affects dendritic maturation and spine morphology, abnormalities that have been attributed to its action in astrocytes [440,441]. AS mice also display disrupted electrical communication between cerebellar PCs and the cortex resulting in abnormal E-I balance [442]. *Ube3A*-haplosufficient mice display only mild impairment of cerebellar function with deficits in learning but not in locomotor function [436]. Consistent with loss of expression in GABAergic interneurons, AS mice display a reduction in the tonic inhibition of granule neurons [436]. In cerebellar PCs, UBE3A-deficiency results in enhanced autophagy through the activation of AMPK and inhibition of mTORC1 [443]. Deregulated E-I balance and autophagy have both been implicated in ASD pathogenesis [444,445].

*Oxytocin (OXT):* Best known for its role in uterine contractions during labor and in lactation, OXT, a nine-amino-acid neuropeptide regulates social behaviors such as including social attachment, social cognition, anxiety, aggression, and immune functioning [446,447,448]. These actions of OXT are mediated through binding to its G-protein coupled receptor. A meta-analysis examining the linkage of OXT with ASD identified multiple SNPs [449]. Other studies have identified genetic variations in the OXT receptor that are associated with ASD [176,449,450,451]. In mice, mutation of the genes expressing OXT or its receptor results in altered circuit connectivity and deficits in social interaction and memory [452,453,454]. OXT expression is altered by alterations in the functioning of other ASD-linked proteins. For example, OXT level is reduced in CNTNAP2, SHANK3, and NLGN3-deficient mice [454,455,456]. Administration of oxytocin and activation of endogenous OXT release via chemogenetic or by pharmacological strategies alleviates social behavior deficits in both mutant CNTNAP2 and SHANK3 mice [454,457,458]. On the other hand, administration of pharmacological activators of NLGN3 signaling in NLGN3-knockout mice restores OXT levels while also alleviating ASD-like behavior [455]. Assuming that the four proteins are part of a common signaling pathway, this puts OXT upstream of Cntnap2 and SHANK3 and downstream of NLGN3.

Although the cerebellum expresses the OXT receptor and it is known that OXT modulates circuitry between the cerebellum and the striatum [459], the function of OXT in the cerebellum is not clear. Within the cerebellum, the OXT receptor is expressed in PCs of lobule Crus I, the dysfunction of which is implicated in ASD. However, blockage of OXT receptor in PCs of lobule Crus I in wild-type mice does not induce autistic-like social, stereotypic, cognitive, and anxiety-like behaviors. Similarly, activation of the OXT receptor in this brain area does not alter the electrophysiological properties of Crus I PCs nor alleviate symptoms in ASD mice [460]. Although this may suggest that OXT action in PCs of Crus1 are not involved in ASD, it is possible that it contributes to but is not sufficient for ASD development. A recent study described that cerebellar damage, which has been associated with ASD, upregulates OXT receptor expression in BG cells much more so than occurs in PCs [461]. 

*Chromodomain helicase DNA-binding protein-2 and -8 (CHD2 and CHD8*): CHD proteins are a family of chromatin remodeling proteins that have been linked to neurodevelopmental disorders [462,463]. Among the CHD proteins, CHD2 and CHD8 are linked to ASD [464]. Although expressed in the cerebellum, both CHD2 and CHD8 are expressed more highly in the hippocampus and cortex. While function of these proteins is unclear, existing information suggests that they promote proliferation of neural progenitor cells. Deletion of CHD8 specifically in proliferating cerebellar granule progenitor cells resulting in reduced proliferation and differentiation of these cells and ectopic localization of PCs with the mice displaying cerebellar hypoplasia, ataxia, and psychiatric symptoms [465,466]. Within granule neuron precursor cells, CHD8 regulates the expression of many genes, including genes linked to ASD [465]. Although CHD8 mutant mice display motor coordination defects, other cognitive or behavioral ASD-associated defects are not observed [465]. 

*Astrotactin 2 (ASTN2):* ASTN2 is a transmembrane glycoprotein that displays the highest expression in the cerebellum, with low levels in the cortex and hippocampus [467,468]. Within the cerebellum, ASTN2 is expressed in PCs and granule neurons regulating synaptic activity and trafficking of receptors and other synaptic proteins [467,468]. Recent research has identified ASTN2 mutations, including CNVs, that cause or increase risk for ASD and other neurodevelopmental disorders [68,469]. *Astn2* knockout mice have altered cerebellar circuitry, abnormal dendritic morphology in Crus1 and posterior vermis, disrupted E/I balance and display ASD-like behaviors [68] indicating that the effect of ASD-linked mutations of ASTN2 is due to loss-of-function. Mice with PC-specific deletion of ASTN2 also display ASD-like behavior underscoring the importance of cerebellar dysfunction in ASD [68].

Table 1 provides a brief description of the proteins encoded by the genes included in the section above along with their primary functions, and major evidence linking their dysfunction/deregulation to ASD.

(B) Whole Genome Analyses

Identification of disease-modifying genes by GWAS and whole-exome sequencing are not reliant on an a priori hypothesis for the development of the disorder. While these unbiases analyses have been productive for many genetically-influenced disorders, a fundamental challenge in understanding the genetic mechanisms underlying ASD is the heterogeneity of the disorder. An estimated 1100 genes are implicated in ASD [470], of which close to 200 have been identified with high-confidence carrying rare de novo and transmitted genetic variants. Further complicating the understanding of the genetic basis of ASD is that over 90% of the high-confidence genes are expressed during early development and most of these are associated with other neurodevelopmental disorders [471,472,473,474]. In children, ASD often presents alongside comorbidities including attention-deficit-hyperactivity disorder (ADHD), intellectual deficiency (ID), and epilepsy [475,476]. In adults, symptoms of ASD are frequently observed in individuals with schizophrenia, bipolar disorder, and other disorders with neurodevelopmental underpinnings [475,476]. In this review, we focus on peer-reviewed publications limited to the past 5 years. While finding the significance of the genes identified in these large-scare studies to cerebellar function has been attempted, information is limited for a majority of the genes described below.

A study of patients with either ASD or ADHD and that used Genomic Structural Equation Modeling (SEM) to GWAS summary statistics combined with transcriptome-wide analysis, identified 83 unique genes with expression changes associated with ASD but not any other closely-linked neurodevelopmental disorders, many of which were novel [477]. Interestingly, many of these ASD-specific genes overlapped with gene sets implicated in bacterial skin disease and erythema. This is consistent with the previously described association between ASD and immune system dysfunction, particularly involving the skin [478,479,480,481]. One limitation of the study is that it only included individuals of European ancestry.

A recent study in which the genetic variation of 91 circulating inflammatory factors was analyzed from genome-wide association studies (GWAS) database of over 18,000 individuals of European ancestry with ASD suggested positive association with ASD of multiple immune system-associated genes, including those of *CD244*, *CD5*, *FLT3LG*, sulfotransferase 1A (*SULT1A*), and TNFSF10, while levels of IL-7, IL2Rβ, and IL-2 were negatively associated [482]. SULT1A is expressed in both neurons and glia in specific regions of the brain with high expression in the cerebellum [483].

A meta-analysis of a GWAS database of 18,000 individuals with ASD (but without other neurodevelopmental disorders), reported the identification of five loci and many common genetic variants that contributed to ASD but not to other disorders. These genes included *CADPS*, *KCNN2*, *KMT2E*, *MACROD2*, *NEGR1*, and *PTBP2* [484]. Of these SNPs in the *PTBP2*, *CADPS*, *KCCN2* and *KMT2E* gene have previously been linked to ASD by GWAS, and as described below, in the case of CADPS, by clinical and experimental research. Interestingly, the *CADPS* and *NEGR1* genes were identified in another recent genetic analysis aimed at identifying genes that are co-linked with ASD and another neurodevelopmental disorder(s) [175]. In this study both *CAPDS* and *NEGR1* were also associated with epilepsy. Furthermore, *NEGR1*, which encodes a neuronal growth regulator, displayed statistically significant genetic association with ASD only in females.

Several studies have described aberrant splicing and genetic variations of the *CADPS2* (calcium-dependent activator protein for secretion 2) gene (which is closely related to CADPS) causes ASD [485,486,487]. Additionally, duplication, deletion, and translocation within the *CADPS2* gene locus have been linked to ASD [488,489,490]. Mice lacking CADPS2 also display ASD-like behavior [486]. CADPS2 is most highly expressed in granule neurons of the developing cerebellum where it stimulates release of BDNF and NT3 from parallel fiber terminals. CADPS2 knockout mice exhibit ASD-like symptoms along with impaired release of BDNF and NT3 leading to delayed cerebellar development along with death of granule neurons and stunted PC branching [491]. CADPS2 is also critically involved in the normal release of oxytocin, a social interaction modulatory protein, from the pituitary [492]. Although CADPS2 levels are high in the hypothalamus and pituitary, its release from the pituitary of CADPS2 knockout mice is low [492].

The *KCNN2* (potassium voltage-gated channel subfamily C member 2) gene encodes SK2, a member of the small-conductance Ca^2+^-activated potassium channel family widely expressed in the brain and localizing to synaptic terminals [493]. By affecting neuronal excitability and signaling, SK2 regulates memory and learning, and in synaptic plasticity [494]. The level of SK2 levels at synaptic terminals is regulated by UBE3A, which inhibits the recycling of endocytosed SK2 back to the synaptic membrane [494,495]. As described above, increased expression of UBE3A increases risk of ASD whereas deficiency causes Angelman syndrome [496,497]. SK2 regulates PC development, and following cerebellar development, SK regulates activity-dependent dendritic plasticity of PCs [498,499,500]. Humans with KCNN2 gene mutations display ataxia, impairment in language development, and intellectual disabilities [493].

KMT2E (Lysine methyltransferase 2E) is a N-methyltransferase that negatively regulates gene transcription by methylation of histone 3 [501]. De novo haplosufficient mutations and variants in the *KMT2E* gene are linked to a variety of neurodevelopmental disorders, including ASD, often accompanied by macrocephaly [502,503,504]. Non-functional variants resulting from truncating mutations or defective splicing in KMT2E produces cerebellar hypoplasia and ASD symptoms [503,505,506]. Prenatal alcohol exposure in mice increases expression of KMT2E in the developing postnatal cerebellum which disrupts proliferation and neuronal differentiation [507].

PTBP2 (Polypyrimidine tract-binding protein 2) is a neuronal splicing factor that plays a crucial role in brain development by regulating neurogenesis through the control of differentiation and maturation of neurons [508,509]. Alterations in splicing have been reported in the ASD brain, although the extent to which PTBP2 dysfunction contributes to it are unknown. While studies linking PTBP2 dysfunction in the cerebellum to ASD are limited, the protein’s critical role in neuronal RNA splicing suggests that deregulation of its function could contribute to abnormal neurogenesis in the cerebellum.

Other genes and loci recently linked to ASD by analysis of summary statistics of previously reported GWAS data are *XRN2*, *NKX2-4*, *PLK1S1*, *CRHR1-IT1*, *C8orf74* and *LOC644172* [510]. Multiple genetic studies have identified the *MACROD2* gene as being linked to ASD [511,512,513,514]. MACROD2 is best known for its role in suppressing colorectal and other cancers [515]. While MACROD2 expression is not altered in the ASD brain, the expression of *RPS10P2-AS1*, a long-coding RNA that is transcribed from a region close to the MACROD2 gene on Chromosome 5, is increased 7-fold in postmortem cortex of ASD patients and regulates development of human neural progenitor cells [516,517,518]. It has been suggested that the GWAS linkage to ASD is likely connected to RPS10P2-AS1 rather than the coding *MACROD2* gene [518].

A large-scale exome sequencing study combining data from about 36,000 individuals, including about 12,000 with ASD, identified 102 ASD risk genes most of which represented de novo variants [519]. Four risk genes (*SLC6A1*, *DEAF1*, *KCNQ3*, and *SCN1A*) that were identified in which mutations confer a gain-of-function effect [519]. Gain-of-function variants in the *KCNQ3* gene, which encodes a K+ channel, have previously been reported to cause ASD [520]. Mutations in the *SCN1A* gene, which encodes the alpha 1 subunit of the sodium channel, causes or is associated with several epilepsy disorders. Of the 102 risk genes, 12 genes were located in regions impacted by copy number variation (CNV). Another gene that has been identified in other studies, *KMT2E*, was also identified as a risk gene in this study. Based on genetic, cognitive, and behavioral criteria used in the analyses, the authors could separate 53 genes as conferring risk predominantly for ASD, whereas the other 49 had broader relevance connecting to both ASD and other neurodevelopmental disorders (NDDs). Most of the 102 risk genes were found to affect neuronal communication or gene expression regulation (GER). Expression analyses in the normal human cortex indicated that the risk genes participated in mid-to-late fetal development and with most being expressed in maturing and mature neurons that were both excitatory and inhibitory [519]. Analysis of the GER genes suggested involvement in the regulation of E/I balance.

Another parent-offspring trio whole-exome sequencing (WES) study conducted in a group of 60 individuals with both ID and ASD, identified variants in previously identified eight ID/ASD-linked genes (*SYNGAP1*, *SMAD6*, *PACS1*, *SHANK3*, *KMT2A*, *KCNQ2*, *ACTB*, and *POGZ*) [521]. Other studies have identified *SYNGAP* as an ASD risk gene [522,523,524]. SYNGAP1 protein (synaptic Ras GTPase-activating protein-1) is present at high amounts in the postsynaptic cells of excitatory synapses [525,526]. Interestingly, although most studied in the context of synaptic function, SYNGAP1 also regulated neurogenesis [527]. Additionally, four novel candidate ASD/ID genes with de novo truncating variants (*MBP*, *PCDHA1*, *PCDH15*, and *PDPR*) were identified [521]. The pyruvate dehydrogenase phosphatase regulatory subunit (PDPR) is variably expressed in the brain, with the highest levels in the corpus callosum and in the cerebellum.

Most whole-genome studies have focused on de novo variants identified from parent-offspring trios. An integrated model composed of a two-stage analysis of de novo and inherited genetic variants, which provides yields greater power to identify risk genes [528], was used to study about 43,000 ASD cases, including ~35,000 new cases identified 60 genes including five new moderate-risk genes (*NAV3*, *ITSN1*, *MARK2*, *SCAF1* and *HNRNPUL2*). These five genes represent rare loss-of-function variants with modest effect size, not strongly associated with ID compared with five of the most common highly-penetrant genes (*CHD8*, *SCN2A*, *ADNP*, *FOXP1*, and *SHANK3*) [529,530]. Findings related to SHANK 3 and CHD8 in ASD are described above. Activity-dependent neuroprotective protein (ADNP) is most highly expressed in the cerebellum and hippocampus, and best known for its neuroprotective activity [531]. In the ASD brain, ADNP dysfunction causes disruptions to DNA methylation and mitochondrial gene expression in the cerebellum [532].

The *SCN2A* gene encodes the Na(v)1.2 channel protein that is expressed highly and selectively in the cerebellum in rodents and non-human primates [533]. *SCN2A* variations in humans cause seizures, ataxia, and cerebellar atrophy [534]. Clinical exome sequencing has identified specific loss-of-function variations in *SNCA*, which carries one of the highest risk for ASD of any gene in humans [519,535]. In mice, ASD-associated SCNA variants disrupt granule neuron to PC communication and impairs neuronal plasticity in the cerebellum [536].

Given the importance of inherited genetic factors, an approach that was used recently to better understand the ASD architecture is the identification of moderate effect size (MES) genes in ASD [537]. MES genes do not qualify as risk genes by themselves but contribute to ASD when paired with specific other MES genes. The first step of the study identified inherited variants in two distinct genes overrepresented in offspring with ASD with non-ASD offspring potentially inheriting either, but not both variants. Analysis of about 11,000 families led to the identification of 97 MES genes forming 50 significant gene pairs [537]. One pair of genes identified as ASD-associated was *SNAP25* (Synaptosome Associated Protein 25) and *SLPI* (Secretory Leukocyte Peptidase Inhibitor). A study using whole genome sequencing of 32 Chinese ASD trios found de novo mutations, inherited variants, copy number variants (CNVs) and genomic structural variants [538]. This study identified 87 potential risk genes from 4832 genes containing variants [538]. Among these were microcephaly-related genes and genes involved in centrosomal function and chromatin remodeling.

A recent transcriptomic analysis of postmortem cerebellar PCs isolated by laser capture and pooled from postmortem ASD tissue identified about 430 differentially expressed genes, which were involved in regulating neurodevelopmental processes and connectivity, organization of the ECM, calcium response, and immune function [105]. Despite the compelling results of this and other genetic studies utilizing both ASD patients and mice, two recent studies analyzing the UK Biobank summary data failed to identify specific subregions of the cerebellum displaying significant genetic correlations with ASD at the whole genome-level, suggesting smaller pleiotropic cerebellar subregions than were analyzed and/or canceling-out of opposing genetic correlations across the genome.

## 6. Non-Genetic Factors That Combine with Genetic Susceptibility in ASD

Recent studies have suggested important roles for alterations in gut microbiota in the development of ASD [114,539]. Both the incidence of ASD and severity of the associated behavioral symptoms correlate with gastrointestinal (GI) disturbances [540,541]. GI disturbances likely result from alterations in the gut microbiota, which can alter gut permeability and promote neuroinflammation. GI alterations and neuroinflammation in ASD has been attributed to changes in serotonin and tryptophan synapse metabolism and the production of neuroactive metabolites [542,543,544,545]. Supporting a causal role for gut microbiome changes in ASD are the findings that germ-free wild type mice transplanted with gut/fecal microbiota from ASD patients develop ASD-like behaviors [542,543,544,546]. In the VPA mouse model of ASD, impairment in social behavior and cognition are associated with microbial alterations and cerebellar neuroinflammation [547,548]. Furthermore, transplantation of gut microbiota from a healthy donor or the administration of a prebiotic diet reverses the microbiome alteration and neuroinflammation along with normalization of the ASD-like phenotype [547,549]. Maternal gut microbiota alterations can also increase the risk of ASD in the offspring [550]. In the BTBR mouse model of idiopathic ASD [551], cerebellar abnormalities, including cerebellar hypertrophy and decreased maturation PCs, are associated with GI changes [552]. These cerebellar alterations and ASD-like behavioral phenotype are normalized by administration of butyrate, a gut microbial metabolite, to the dam [550]. The genetic mechanism by which gut microbiome changes contribute to the psychiatric issues in ASD are not known. One candidate gene might be SHANK3, which besides being expressed in various regions of the brain is also expressed in the GI epithelium [553,554]. SHANK3 knockout mice, which display an ASD-like phenotype along with cerebellar abnormalities, have a significantly altered microbiota composition [553,554].

Dysregulation of the immune system is another factor commonly associated with ASD [115,555]. A bioinformatic study identified bacterial lipopolysaccharide and immune-related (BLI) gene signatures in ASD. [556]. In mice, immune system dysfunction has been described to be associated with cerebellar abnormalities. *En2*-knockout mice, which display cerebellar defects, display reduced levels of pro-inflammatory cytokines in the cerebellum but elevated levels peripherally [338]. Similarly, SHANK3b knockout mice (lacking the 3b isoform of SHANK3 and that display an ASD phenotype) also show dysregulation of the immune system in the cerebellum and peripherally through adulthood [557,558].

Maternal immune activation (MIA), generally caused by infections, also increases the risk of ASD [115,559,560]. MIA-induced ASD can be modeled in mice by gestational administration of the viral mimic polyinosinic: polycytidylic acid (poly I:C) [561,562]. Offspring of MIA-mice display decreased number of cerebellar PCs, particularly in Lobe VII [563,564]. The loss of PCs is accompanied by increased expression of Nox1/NADPH, an enzyme that generates reactive oxygen species [565]. MIA-mice lacking NOX1 display a reduced loss of PCs and an ameliorated ASD phenotype [565]. Supporting a role for elevated oxidative stress in the ASD brain is the finding of substantially reduced glutathione (an endogenous anti-oxidant) in the cerebellum of individuals with ASD [566]. Another study described increased levels of specific neuroinflammatory cytokines, reduced expression of the cerebellin-1 and GluRδ2 synaptic proteins in the cerebellum along with deformed synapses [564]. Loss of PCs and ASD-like behavior is also observed in dams infected with influenza virus, supporting a role for MIA in ASD [567]. Offspring of mice injected mid-gestationally with VPA display chronic glial activation in the cerebellum and hippocampus through adulthood [568].

In sum, gut microbiome alterations, immune system activation, and MIA are associated with ASD and rodent models have been used to study the connections of these alterations to the disorder. How these factors interface with dysfunction of the brain, and specifically the cerebellum, remains to be resolved but it is likely that they act in combination with genetic susceptibility. Identifying the genetic linkages will lead to the development of effective targeted diagnostic and therapeutic strategies.

## 7. Conclusions

Much progress has been made in our understanding of ADS since the time it was referred to as “infantile psychosis” and blamed on a cold and emotionally distant “refrigerator mothers” [569,570]. Despite this, our understanding of the molecular and cellular underpinnings of ASD remains limited. The phenotypic and genetic heterogeneity of ASD and its frequent co-occurrence in other neurodevelopmental and psychiatric disorders has made it challenging to identify specific genes that are causally involved in the development of the disorder. As our review describes, however, compelling evidence from human genetic analyses and experimental studies conducted in rodents point to a key role for cerebellar dysfunction in the development and progression of ASD. Although most attention has been placed on PCs, accumulating evidence suggests that dysfunction of granule neurons, other neuronal types, and glial cells, as well as disruption in the normal interactions between PCs and these other cell types, contribute to ASD. Cerebellar dysfunction impacts the functioning of other brain regions connected to by the cerebellum. Besides synaptic dysfunction, the identification and analyses of ASD-linked genes has identified deregulation of neurogenesis as a common cellular feature of ASD. Recent studies have suggested important roles for alterations in gut microbiota [114,539], innate immune system dysfunction [115,555] and maternal inflammation [115,559,560] in the development of ASD. How these factors interface the cerebellum and other parts of the brain to cause ASD remains to be resolved.

## Figures and Tables

**Table 1:** Summary of proteins associated with ASD along with their primary functions.

Protein	Functional Class	Primary Function	Association with ASD and Potential Mechanisms
**SHANKs**	Synaptic	Postsynaptic scaffolding proteins at glutaminergic synapses.	- ASD-linked LOF mutations/polymorphisms in all three SHANK genes in humans. - SHANK1 LOF mice display ASD phenotype and reduced mGluR1 signaling. - SHANK2 LOF mice display ASD phenotype and reduced GABA-A receptor function and disrupted E/! balance. - SHANK3 LOP display ASD phenotype, altered dendrite and spine morphology, deregulated glutaminergic synapses and disrupted E/I balance.
**Neuroligins (NLGNs)**	Synaptic	Postsynaptic transmembrane proteins regulating synapse formation, organization, and function.	- ASD-linked LOF mutations/polymorphisms in all four NGLN genes in humans. - KO of each of the four NLGN genes in mice produces ASD phenotype. - KI of human ASD mutations in NLGN markedly reduces its stability along with impaired synapse elimination, E/I balance, and inhibitory cerebellar output to other brain regions. - NLGN4 is required for organization and functioning of glycinergic synapses. KI mice show impaired NLGN4 cellular localization and dendritic spine formation, and display ASD phenotype.
**Neurexins (NRXNs)**	Synaptic	Presynaptic transmembrane cell adhesion proteins that regulate synapse formation, organization of release machinery, and E/I balance.	- Genetic variants of all three NXN genes are associated with ASD in humans - NRXN1 and NRXN2 KO mice display ASD phenotype. - NRX1 KO mice display electrophysiological abnormalities. - NRXN2 KO mice display synaptic abnormalities. - NRXN3 regulates glutamate and GABP release in a brain region-specific manner. Deletion of some isoforms impairs dendritic synapses.
**Contactin-associated protein-2 (CNTNAP2)**	Synaptic	Cell adhesion molecule involved in synapse organization. Also involved in the clustering voltage-gated K+ channels myelinated axons at Nodes of Ranvier.	- Mutations are linked to ASD in humans. - KO mice display reduced cerebellar volume, altered PC morphology in Crus I/II regions of the CBM. - PCs display electrophysiological impairment in response to somatosensory stimulation. - Within the cortex (CASPR2) is involved in the regulation of neuronal migration, glutamate receptor organization, and morphology of dendritic arborization and synaptic spines.
**Cadherins (CDHs)**	Cell adhesion	Involved in the formation of neural circuitry and regulation of synaptic plasticity.	- Mutations in several Type-II cadherin genes linked to ASD in humans. - Many cadherins are expressed during the development and maturation of the CBM. - CDH 9 and 11 expression in PCs decreases during maturation of the CBM. - CDH 13 is expressed in Golgi cells of the CBM. KO mice display some ASD features. - Atypical CDHs, FAT1 and Celsr3 are expressed highly in the CBM. - FAT1 expression is reduced in human ASD iPSCs. Mice lacking Celsr3 display dendritic abnormalities and some ASD features.
**CUB and sushi multiple domains-3 (CSDM3)**	Synaptic Development	Regulates dendrite development and synaptogenesis.	- Mutations in the CSDM3 are linked to ASD in humans. - CSDM3 KO mice display ASD phenotype. - CSMD3-deficient mice display abnormal PC morphology in Right Crus I/Crus II lobules along with E/I imbalance in PC synapses.
**Engrailed-2 (EN-2)**	Development	Transcription factor regulating pattern formation that is expressed highly in the cerebellum and hindbrain.	- Necessary for development of the CBM.
**Tuberous sclerosis complex protein-1 and 2 (TSC1/TSC2)**	Tissue homeostasis	Act as part of a complex to inhibit mTOR signaling. TSC1/TSC2 mutations cause tuberous sclerosis complex.	- A high proportion of patients with mutant TSC1/TSC2 mutations induced TSC in humans have ASD. - Mice lacking either TSC1 or TSC2 display PC loss, cerebellar dysfunction and ASD symptoms. - mTORC1, which is inhibited by TSC1/TSC2, and its target eIF4E are dysregulated in mouse models of ASD along with reduced PCs.
**PTEN**	Tumor suppressor	Lipid phosphatase that negatively regulates cell division and survival.	- Low PTEN levels or PTEN mutations are linked with ASD with microcephaly. - Required for normal development of the CBM. - PTEN KO mice display structural and functional PC abnormalities and exhibit ASD-like behavior.
**BMAL1**	Transcription factor	Critical component of the circadian rhythm regulatory machinery. Also regulates mRNA translation.	- Disrupted circadian rhythm is a common feature of ASD in humans. - KO mice have abnormalities in CBM cell densities and dendritic structure, and display ASD phenotype. - BMAL1 PC-specific deletion of BMAL in PCs produces ASD phenotype.
**Retinoic acid receptor-α (RORα** **)**	Nuclear receptor	Multiple roles. In the CNS, RORα regulates stem cell production, neuronal differentiation, and morphogenesis of brain structures.	- Regulates synaptic plasticity and E/I balance. - Highly expressed in PCs and required for PC survival. - KO mice display multiple abnormalities in PC structure and function and develop cerebellar dysfunction.
**UBE3A**	E3 ubiquitin ligase and transcriptional regulator for steroid hormone receptors	Regulates synaptic function and plasticity	- Reduction or complete loss of UBE3A gene expression causes Angelman syndrome in humans (disorder with ASD features). - Highly expressed in the cerebellum. - Mice with Ube3A deficiency display impaired cortico-cerebellar communication and abnormal E/I balance.
**Oxytocin (OXT)**	Neuropeptide	Uterine contractions.	- Regulates social, cognitive, and immune functioning in humans. - Multiple SNPs are associated with ASD in humans. - Expressed in PCs within Crus I region.
**Chromodomain helicase DNA-binding protein-2 and 8 (CHD2/CHD8)**	Chromatin remodeling protein.	Regulates transcription, DNA replication, and DNA repair by controlling chromatin structure and accessibility.	- Mutation of both CHD2 and CHD8 are linked to ASD in humans. - CHD8 KO mice in proliferating granule cell precursors affects production of granule neurons and causes ectopic PC localization.
**Astrotactin-2 (ASTN2)**	Transmembrane glycoprotein	Regulates trafficking and degradation of cell surface proteins.	- Mutations cause or increase risk of ASD in humans. - KO mice display altered CBM circuitry along with abnormal dendritic morphology in Crus I and disrupted E/I balance.

Proteins described in this review are listed along with their primary function and major evidence of their association with ASD. Unless indicated otherwise, evidence presented in the table comes from rodents. Evidence described comes largely from studies of the cerebellum (CBM). PC refers to Purkinje cells, E/I to excitatory/inhibitory, KO and KI to knockout and knock-in, and LOF to loss-of-function.
